# Lest we forget: Dr Wu Lien-Teh (1879–1960)

**DOI:** 10.1177/09677720231177679

**Published:** 2023-06-04

**Authors:** Anoushka Bucktowar, Hareesha Rishab Bharadwaj, Matan Bone

**Affiliations:** 1 School of Medical and Dental Sciences, 1724 The University of Birmingham, Birmingham, UK; 2 Faculty of Biology Medicine and Health, 5292 The University of Manchester, Manchester, UK

From orchestrating the creation of the Wu mask, which would serve as the precursor of the current N95 mask, to revolutionising China's public health and safety systems, Dr Wu Lien-Teh (1839–1960) represents one of the most influential figures in modern medical history. His story, one which would witness a young son of Chinese immigrants to the Straits Settlements, presently Malaysia and Singapore, rise to the ranks of a public health champion, transverses countries, continents, and cultures; it is this very unique journey that the authors aim to narrate through this piece.

Born in Penang, which was then under British colonial rule, Wu received his early education at the Penang Free School, a Church of England School.^1^ In 1896, he won the Queen's Scholarship which opened his doors to studying medicine at Emmanuel College, Cambridge, becoming the first student of Chinese descent to ever attend. He was an outstanding performer at university, winning almost all the available scholarships and prizes.^2^

He quickly developed a firm reputation in the medical academia, travelling to Liverpool, Paris and Germany for research. Wu returned to the Strait Settlements in 1903, where he joined the Institute for Medical research in Kuala Lumpur, spending his early medical career researching roundworms and beriberi.^3^

**Figure 1.**
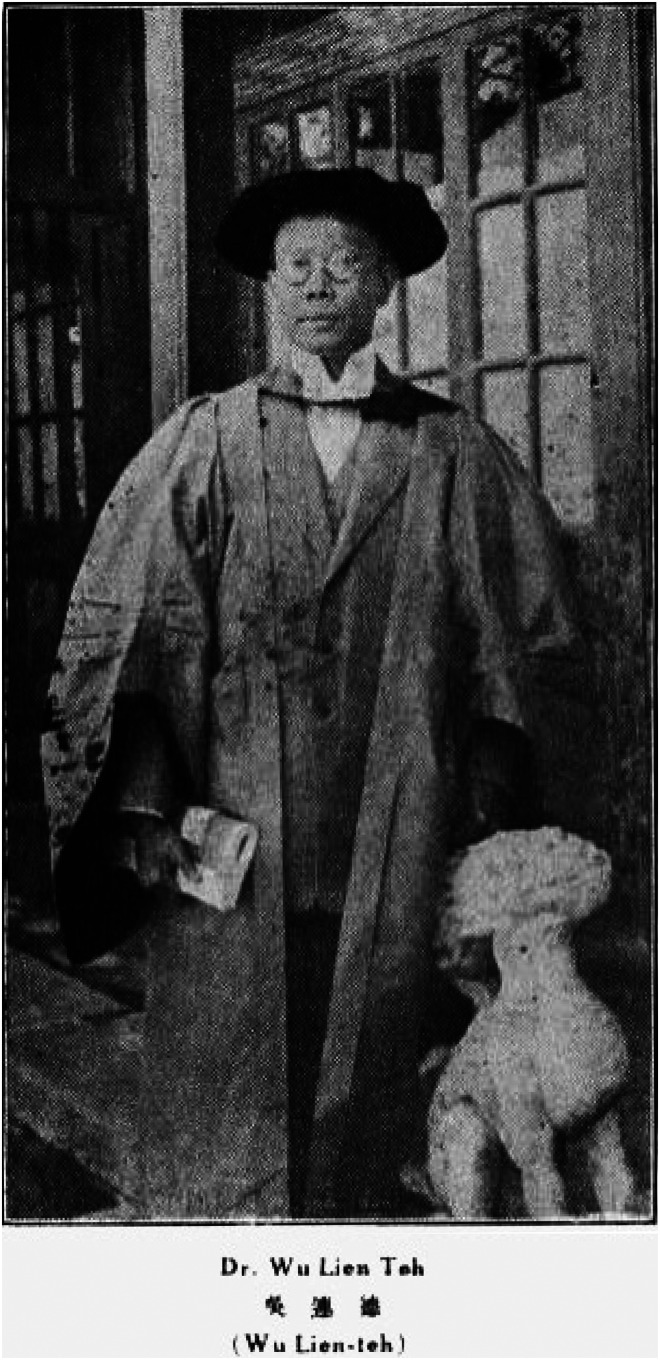
Dr Wu Lien-Teh^4^.

Dr Wu is best recognised for his contributions in combatting the Manchurian plague.^1^ This invariably fatal epidemic broke out in the Chinese North-Easterly region in the winter of 1910, spreading rapidly from Manchouli to Harbin. Wu was summoned to Harbin to investigate this unknown disease which had claimed the lives of 40,000 people in just 4 months. Here, he performed the first-ever post-mortem examination in China, on a Japanese woman who was a victim of the plague, despite strong opposition against this practice. The general consensus was that the disease was being transmitted by fleas or rats. However, Wu discovered in body tissues that the causative agent was *Yersinia pestis*, the same bacteria that had caused the Black Plague centuries ago. This was a pneumonic plague which was transmitted via human sputum or breath (Figure 1). This finding was met with extensive disbelief among his scientific colleagues.^1^

At the time of his discovery, Wu knew exactly what he was looking at. Quoting from an obituary of Wu published in the *British Medical Journal*: ‘He had no doubts what he was up against. At the time of diagnosis, the pneumonic plague was a sentence of death. Doctors stood in front of their patients in full blast of their breath to examine their chests. They paid the price’.^5^

**Figure 2.**
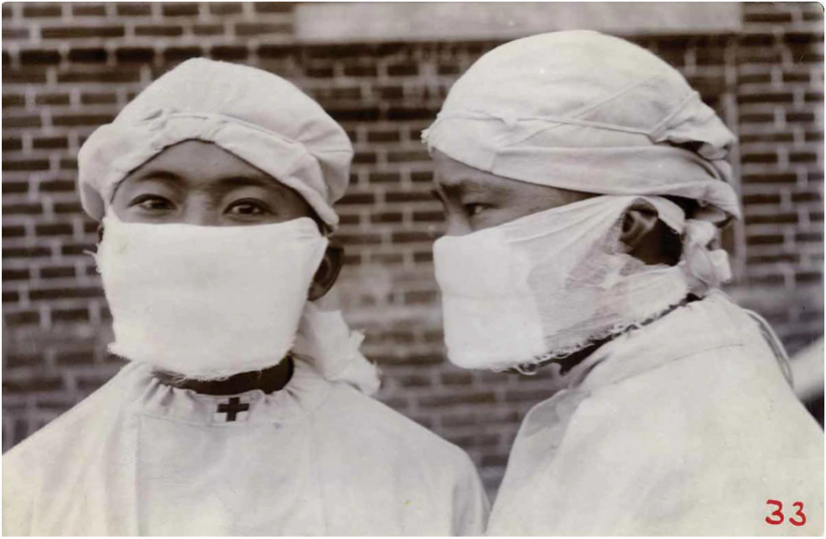
Pictorial representation of Dr Wu's masks^6^.

To protect against the transmission of the airborne disease, Wu attempted to design a more protective tool than the previously known anti-plague masks. He added more layers, more gauze and came up with better ways to tie the mask to prevent entry of the germ. This revolutionary design is presented as Figure 2, a pictorial representation of Dr Wu's masks.^6^ Today, his invention is recognised as a precursor to the modern N95 masks, which are widely used in modern medical practice, and which were the cornerstone of staff protection during the COVID-19 pandemic.

However, there was more to be done. Wu was shocked to find thousands of unburied bodies of infected victims in the area. He knew that once the seasons got warmer, these bodies would pose a serious public health danger by facilitating the rapid spread of the disease. He insisted and convinced local officials to perform a mass cremation, despite substantial cultural opposition against the practice.^1^ At the same time in 1911, Wu persuaded Japanese and Russian railway authorities to halt train operations to prevent transmission of the disease across North-Eastern China and beyond. These measures were monumental in containing the Manchurian plague. By the time, the Chinese New Year came, the disease had run its course.

In April 1911, the National Plague Conference was held in Mukden, including internationally respected epidemiologists from 11 countries (including USA, UK, Japan, France, and Russia) in attendance. Wu was elected to lead the conference as President. He was commended by all for his instrumental work in controlling the epidemic. Soon after the conference, China began embracing modern medical science; Wu focused a lot of his efforts in founding the Chinese Medical Society, and establishing medical institutes across China.

In 1935, Wu became the first person of Chinese descent to be nominated for a Nobel Prize in Physiology and Medicine. In his later years, he earned several honorary degrees and awards, and contributed extensively to developing medicine in China. At the age of 81, Wu died from a stroke in his home in Penang, after having published an autobiography in English: ‘Plague Fighter: The Autobiography of a Modern Chinese Physician’.^2^

**Figure 3.**
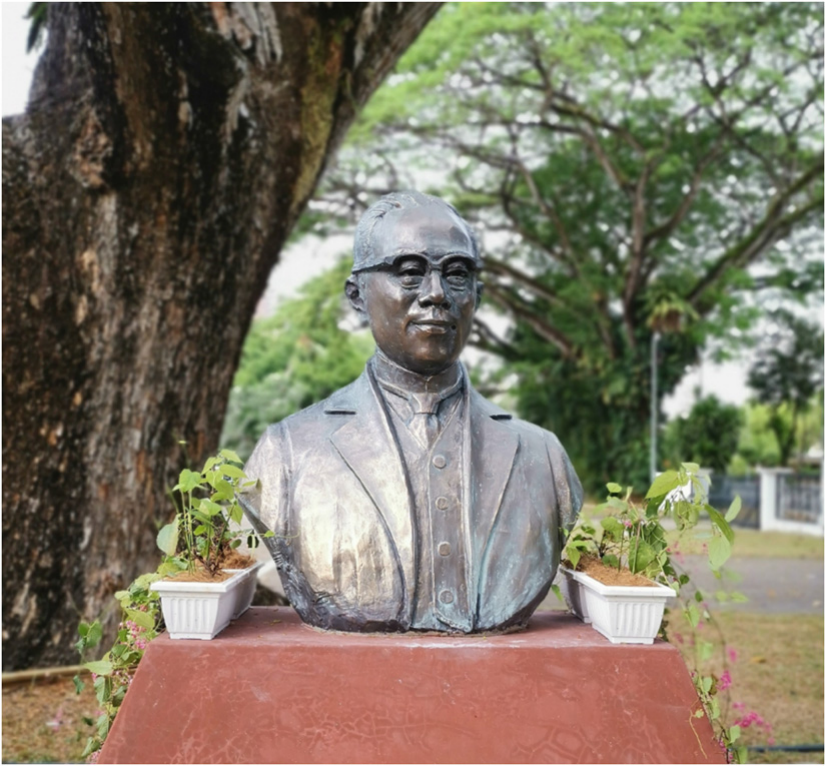
Statue of Dr Wu Lien-Teh located at the Penang Institute^7^.

The Dr Wu Lien-Teh society has also been founded in Penang to commemorate his lifetime contributions to public health.^7^ The society actively supports activities such as lectures, conferences and workshops to promote his legacy. We thank the society for their monumental role in spreading Dr Wu Lien-teh's influential life journey through their many activities and endeavours (Figure 3).

Wu's pivotal impact on healthcare provision in China led to the establishment of the Wu Lien-Teh Institute at the Harbin Medical University in 2015. In 2019, the renowned journal *The Lancet* initiated the Wakley–Wu Lien-Teh prize in the honour of Dr Wu and Thomas Wakley, the journal's founding editor. Recently, Wu's work in epidemiology has been extensively utilised during the COVID-19 pandemic, with a drastic increase in the use of N95 masks. However, most of the world learnt of with Dr Wu's work in March 2021 when Google Doodle honoured his legacy by depicting him assembling and distributing surgical masks to reduce infection transmission.
